# Data on the factors driving the decision of rural people to move into the city

**DOI:** 10.1016/j.dib.2024.110037

**Published:** 2024-01-11

**Authors:** Shamima Akhter, Sadika Haque, Mahmuda Nasrin

**Affiliations:** Professor, Department of Agricultural Economics, Bangladesh Agricultural University, Mymensingh 2202, Bangladesh

**Keywords:** Driving factors, Migration decision, Probit model, Household's perception, Job opportunities

## Abstract

The data set explores the driving factors of migration of rural people to the cities. Primary data were collected purposively from 172 farm households from three upazilas of kishoreganj district in Bangladesh. Among 172 households, 89 households had at least one migrant member and 83 households were without any migrant member. Probit model was used to analyze factors of migration decision at the household level. Data set reveals that various factors motivate the decision of the farm households for their member to move into the city. Among which household head age, number of active male member in the family and value of the household asset holding significantly influence migration decision. Beside econometric analysis, household's perception on different motivating factors of migration was also assessed. Most of the households perceived that too many family members, poor living condition, migrant's family influence and job availability in the city mostly motivate the people for migration into the city along with other driving factors.

Specifications Table

**Table:** 

Subject	Economics
Specific subject area	Agricultural Economics
Type of data	Table, figure
How the data were acquired	Field survey using structured questionnaire (included in the supplementary materials)
Data format	Raw, Analyzed
Description of data collection	Farm households are purposively chosen including households with migrant and without migrant member.
Data source location	The field survey was conducted at Kishoreganj district in Bangladesh covering three upazilas. These are Karimganj, Tarail and Nikli upazillas. Link for Fig. 1 : http://www.bbs.gov.bd/site/page/ef4d6756-2685-485a-b707-aa2d96bd4c6c/Vital-Statistics .
Data accessibility	Data is available within this article. DOI:10.17632/mm65dh657w.1 https://data.mendeley.com/datasets/mm65dh657w/1

## 1 Value of the Data

• This data will help to explore the motivating factors of farm household's migration decision to cities in particular region of Bangladesh. Households’ perception regarding migration of one or more of family members is also assessed from this dataset.
• The factor analysis can be used for further research related to regional planning especially urban development planning in response to migration of people from rural to urban areas.
• The data on the migration decision of the households can be helpful for other researchers to apply advance statistical analysis.
• This dataset will be helpful for the policy maker to reformulate the policies regarding internal migration process of the country so that migrant from farm families either can accrue the benefits from migration or they can decide to stay in the village.

## 2 Data Description

**Fig. 1:**
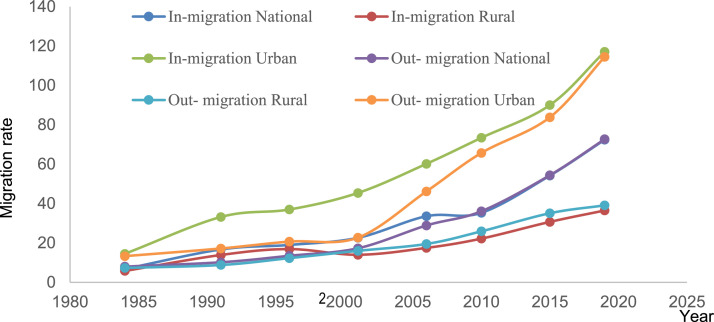
In migration rate and out migration rate per 1000 population in Bangladesh, 1984-2019.

Source: Author's computation based on SVRS [1] and SVRS [2] data.

The data explain the driving factors behind the decision of the farm households regarding migration of their family member to the cities using empirical model. Beside this, different push and pull factors based on household's perception are also revealed by this data set. Fig. 1 shows the in and out migration rate in Bangladesh over the years. Table 1 describes variables used in the empirical model. Descriptive statistics of the variables used in probit model are shown in Table 2. Different factors influencing migration decision of the farm households have been presented in Table 3. Table 4 presents the household's perception on the different pre - assumed factors of migration from rural area to the cities. The survey questionnaire and the data used for this article are uploaded in the data repository. The survey questionnaire included the questions related to the individual characteristics of the respondents and his or her family members as well as different socioeconomic aspects of the households. The dataset contains both numerical and continuous data which can be used for various econometric analyses. Data are available in excel format in the data repository provided with this article.

**Table 1:** Description of variables used in the empirical model.

Variables	Description	Units of measurement
Age of household head (years)	Household's head age in years.	Years
Number of active male	Number of economically active males in the households whose age is between 15-59 years.	Number of persons
Number of active female	Number of economically active males in the households whose age is between 15-59 years.	Number of persons
Very young dependents (Number)	Number of dependent members in the households whose age range between 0 to 5 years.	Number of persons
Young dependents (Number)	Number of dependent members in the households whose age range between 6 to 14 years.	Number of persons
Old dependents (Number)	Old dependents are considered as members of the households between age 60 and above.	Number of persons
Household head education	Education level attained by the household head.	Number of schooling years
Farm size (Hectare)	Farm size is the amount of land occupied by the households.	Hectare
Value of asset holding (BDT)	Value of different assets owned by the households.	Bangladeshi Taka
Value of livestock (BDT)	Value of livestock animals owned by the households.	Bangladeshi Taka
Household debt (BDT)	Household debt is the amount of credit taken by the sample households.	Bangladeshi taka

Source: Author's specifications.

**Table 2:** Socio-demographic characteristics of the farm households with and without migrant member.

Variables	Households with migrant		Households without migrant		
	Mean	Standard deviation	Mean	Standard deviation	T test
Age of household head (years)	53.43	11.32	47.82	13.31	2.98**
Number of active male	2.39	1.02	1.72	1.00	4.32***
Number of active female	1.80	0.91	1.61	0.83	1.36
Very young dependents (Number)	0.47	0.74	0.47	0.65	0.02
Young dependents (Number)	1.56	1.26	1.64	1.31	0.40
Old dependents (Number)	0.54	0.65	0.37	0.62	1.70*
Farm size (Hectare)	0.56	0.41	0.58	0.39	0.41
Value of asset holding (BDT)	46632.58	48308.76	32978.92	29828.50	2.21**
Value of livestock (BDT)	53067.42	58281.23	53122.65	51160.63	1.19
Household debt (BDT)	22078.65	39541.47	16253.01	21672.64	

*Notes:* *, **, *** significant at 10%, 5%, and 1%, level respectively; BDT = Bangladeshi Taka.

Source: Authors estimation.

**Table 3:** Probit model estimates on drivers of migration at the household level.

Variables	Migration decision			
	coefficient	P>Z	Marginal effect	P>Z
Household head age	0.021**(0.109)	0.056	0.007**	0.048
Number of active males	0.399***(0.108)	0.000	0.134***	0.000
Number of active females	−0.003(0.129)	0.982	−0.001	0.982
Very young dependents	0.073(0.195)	0.707	0.025	0.707
Young dependents	0.079(0.101)	0.437	0.026	0.434
Old dependents	0.204(0.201)	0.311	0.068	0.306
Household head education	−0.135(0.102)	0.188	−0.045	0.182
Farm size	−0.182(0.273)	0.505	−0.060	0.503
Value of asset holding	0.000005*(0.000003)	0.101	0.0000018	0.0000011
Value of livestock	0.000002(0.000002)	0.387	0.0000005	0.0000006
Household debt	0.000004(0.000003)	0.243	0.0000015	0.0000013
Constant	−1.94***(0.704)	0.006	-	-
Model summary				
	LR chi2(11) Log likelihood Probability> chi2 Pseudo R2		35.13	
			−101.551	
			0.0002	
			0.148	
	Number of observations		172	

*Notes:* Figures in parentheses are standard errors; *, **, *** significant at 10%, 5%, and 1%, level respectively

Source: Author's estimation

**Table 4:** Farm household's perception on influencing factors of migration.

Factors	Frequency of response	Farm household's response (%)
**Demographic**		
Too many family members	34	38
**Social**		
Unemployment at slack period in the rural areas	23	26
Better education	5	6
Attraction to the city	9	10
Migrant family member and relatives influence	55	62
**Economic**		
Poor living conditions	43	48
Inadequate farm land	31	35
Failure to repay NGO loan	4	5
Lower return from farming	25	28
Lower wage rate in the rural area compared to urban area	30	34
Job opportunity in urban areas	67	75
**Environmental**		
Crop failure due to natural disaster	6	6.74

Source: author's estimation.

The terminologies used for the variable name in different sheet of the excel file are elaborated in one of the excel sheets named as ‘variable definitions’. Here, HH used as everywhere as household. Sheet one in excel file contains the basic information of the respondents who provide us data which includes age, gender, education and occupation. In sheet two, household's migration status, number of family members, age, gender, education, occupation of the family member of the respondents are included as variables. Sheet three consists elaboration of code of different variable used in analysis presented in different excel sheet. Sheet four, named as land holding data, consists of different categories of land occupied by the respective households from which the variable ‘farm size in hectare’ are calculated for the probit model analysis. Excel sheet five represent asset data from which the variable ‘value of household asset’ is calculated. Here, asset data includes the values of different household assets in terms of Bangladeshi Taka (BDT). In this sheet, total value of the livestock animals occupied by the household is also presented. Excell sheet six contains data on household debt. Houshold debt is the amount of Taka which is taken by the household from different sources for various purpose. Value of household debt is taken as an indeppendent variable in the probit model analysis. Data on the factors of migration are presented in excell sheet eight. Table 3 presents this analyized data of factors of migration.

In the excell sheet ten, each factor is defined under the heading of variable definitions.

## 3 Experimental Design, Materials and Methods

### 3.1 Survey area and sampling procedure

The dataset covers three upazilas of Kishoreganj district of Bangladesh. To identify the sample farm households, multistage sampling procedure was followed. Firstly, Dhaka division was selected among the 8 divisions of the country purposively as Dhaka is one of the mega cities in the world and people from every part of the country are rushing to Dhaka city. In the second stage, one district namely, Kishoreganj from the Dhaka division was purposively chosen based on the migration situation and farming practices of the areas. Three upazilas from the selected district were chosen again considering the migration situation. Finally, a total of 172 farm households from different villages were interviewed for collecting primary data. Among them, 89 households had at least one migrant member and 83 households had no migrant member either internally or internationally. The field survey was conducted during February to April 2020. A farm holding is defined as an agricultural production unit having cultivated land either by their own land or through renting.

### 3.2 Data analytical techniques

Different descriptive statistics like sum, average, percentages were used to illustrate the household's perception on migration of one or more of their family members to the cities. Limited dependent variable model (Probit model) was used to analyze the determinants of households’ migration decisions at the farm household level. This model was specified because migration decision is a selection variable and the answer is dichotomous. Migration decision is a dummy variable using the value of 1 as households making decision for migration that is households with migrants and 0 for households without migrants which means the household has no migrant member.

Following [4], the probit model can be written as

(1) $$\text{Pr}\left(M_{i}=1/x_{i}\right)=\Phi \left(\alpha X_{i}\right)=\Phi \left(\alpha 1X_{1i}+\alpha 1X_{2i}+\ldots \ldots \ldots \ldots \ldots \ldots ..+\alpha_{k}X_{\text{ki}}.+e_{i}\right)\ldots \ldots$$

Where Pr is the probability; Mi is the dependent variable, which represents the ith the household's migration decision and binary in nature;

The dependent variable can be expressed as follows:

Mi = 1 if household occupied migrant member or members
Mi = 0 Otherwise
Φ is the cumulative distribution function (CDF) of the standard normal distribution. X_1i_….
X_ki_ are different socio- demographic and economic characteristics of the ith the household influencing household's migration decision α_1_…… α_k_ are the coefficients estimated typically by the maximum likelihood estimation procedure. The error term e_i_ is assumed to be independently and normally distributed with zero mean and variance σ^2^.

Fig. 1 has been computed based on the secondary data on in-migration and out-migration rate per 1000 population of the country at the rural, urban and national level. Here, Microsoft Excel software was utilized. Table 2 represents the data on the socioeconomic characteristics of the farm households with and without any migrant member. The estimations have been carried out by using the software SPSS 20. Here mean and standard deviation have been calculated against each of the variable for two categories of farm households as we have mentioned already farm households with migrant as well as farm households without any migrant member. Mean is found by taking the sum of the observations for each of the variables dividing by their number. In the present case, number of observations is 172 among which 89 for farm household with migrant and 83 for farm household without any migrant member. Standard deviation is calculated by taking the square root of the variance where variance is calculated from the square root of the observations. It measures the dispersion of dataset relative to its mean value. T-test was performed to see that whether the two sets of data are significantly different from each other. T-test uses means and standard deviation of two samples to make a comparison.

### 3.3 Factors of migration

Age of the household head is expressed in number of years. Here, active males and females are considered as those members in the households whose age is between 15-59 years and members aged 60 years and above are considered as old dependent members [3]. Very young dependent members are those members of the households whose age range between 0 to 5 years while young dependents are considered as 6 to 14 years of old. The farm size is the amount of land in hectares owned by sample farm households. This farm size includes the households homestead area, own cultivable land, pond area, mortgaged in land and fallow land owned by them and a deduction of mortgaged out land. Detailed calculation is available in excel sheet 4 with the raw data availability. The variable ‘value of asset holding’ includes the value of different assets owned by the households expressed in Bangladeshi Taka. These assets include television, mobile phone, electric fan, refrigerator, chair table, motor cycle, bicycle, sewing machine and agricultural machineries. Detailed calculation is available in the data sheet named as asset data. Value of livestock also expressed in Bangladeshi Taka which includes the monetary value of different livestock animals owned by the sample households. And finally household debt is the amount of total debt taken by the particular sample households. Table 3 presents estimated data on determinants or drivers of migration at the farm household level. Here Probit model was estimated using software STATA 11.

The estimated probit model is significant (LR chi^2^ (11) = 35.13; Log likelihood = −101.551; Probability> chi^2^=0.0002) and therefore the use of this model for exploring the driving factors of migration of rural people at the household level is relevant. After estimation of probit model, marginal effects are computed separately. In the linear regression model, the regression coefficients can be interpreted as marginal effects. But in non-linear regression models, like the probit model, coefficients cannot be interpreted as marginal effects. The marginal effect of a explanatory variable is given by the partial derivative of the expected value of the outcome variable with respect to the expalanatory variable [5]. Here each of the variables used in the model is described in the above section with Table 1.

Table 4 presents other influencing factors of migration perceived by the farm households besides the above-discussed econometric results of drivers of migration decision. People from the rural corners of the country migrate to the urban areas because of many reasons including both macro and micro level factors representing economic, social and catalytic realities.These factors are categorized according to four different features like demographic, social, economic and environmental factors. Among demographic factors, too many family members imply that the large number of family members who are living together share their income for household expenditure. Social variables include the better education facilities in the city, unemployment in the rural areas at a particular time here mentioned at slack period, migrant's attraction to the city and influence of the relatives those who are already living in the city.

Economic variables included poor living condition of the households with migrant, their inadequate farm land and lower return from farming, failure to repay Non-Government Organization (NGO) loan, and low wage rate in the rural areas . Job availability in the city compared to the rural areas is another economic factor influencing people to move into the city.

Natural disaster and crop failure are also perceived by the farm households as reasons behind their movement to the city. All these economic social, demographic and environmental factors are calculated using descriptive statistical techniques using software SPSS 20. Here, frequency of the response is the number of respondents answer about the factors as a reason for the movement of their members to the city. And then percentage of the response has been calculated dividing the frequency of response by the number of farm households with migrant (89) and multiplied by hundred.

## Ethics Statements

This research is based on the social science data not on the physical science or not on any particular animal. Individual households were well informed about the research objectives and then they willingly provide their data and cooperate with us. Data has been collected with funding from the Bangladesh Agricultural University Research System (BAURES). This institution has already monitored as well as ethically approved the whole project.

## CRediT authorship contribution statement

**Shamima Akhter:** Conceptualization, Methodology, Investigation, Data curation, Formal analysis, Writing – original draft. **Sadika Haque:** Conceptualization, Methodology, Writing – review & editing. **Mahmuda Nasrin:** Visualization, Validation, Investigation.

## Data Availability

Data on the factors drive the decision of rural people to move into the city (Original data) (Mendeley Data)

## Appendix Supplementary materials

Image, application 1

Image, application 2

Image, application 3
