# Supplementary material for: Data on the factors driving the decision of rural people to move into the city
Source: Data Brief. 2024 Jan 11;53:110037. doi: 10.1016/j.dib.2024.110037 (PMC10838684; doi:10.1016/j.dib.2024.110037)
Supplement: Supplementary file 1 [file mmc1.docx]

Akhter, Shamima (2023), “Data on the factors drive the decision of rural people to move into the city”, Mendeley Data, V1, doi: 10.17632/mm65dh657w.1
