# Supplementary material for: Data on the factors driving the decision of rural people to move into the city
Source: Data Brief. 2024 Jan 11;53:110037. doi: 10.1016/j.dib.2024.110037 (PMC10838684; doi:10.1016/j.dib.2024.110037)
Supplement: Supplementary file 3 [file mmc3.doc]

|  |  |  |
| --- | --- | --- |

SL.NO. Questionnaire on

Factors drive the decision of rural people to move into the cities

1. Respondent’s Information

| 1. | Designation of the respondents | 1=Household head ,2= Other member of the household |  |
| --- | --- | --- | --- |
| 2. | Gender | 1= Male,2= Female |  |
| 3. | Household Status | 1=Migrants household, 2= Non- migrants household |  |
| 4. | Name of district | Kishoreganj | |
|  | Name of the Upazilla |  | |
| 5. | Experience in farming |  | |
| 6. | Occupation |  | |

**1. Households socio-economic Information**

- 1. Household Characteristics

| SL  No | No. of family Members | Gender | Age  (year) | Education | **Occupation** | | | |
| --- | --- | --- | --- | --- | --- | --- | --- | --- |
|  |  |  |  |  | Main | subsidiary | | |
|  |  |  |  |  |  |  |  |  |
|  |  |  |  |  |  |  |  |  |
|  |  |  |  |  |  |  |  |  |
|  |  |  |  |  |  |  |  |  |
|  |  |  |  |  |  |  |  |  |
|  |  |  |  |  |  |  |  |  |
|  |  |  |  |  |  |  |  |  |
|  |  |  |  |  |  |  |  |  |
|  |  |  |  |  |  |  |  |  |
|  |  |  |  |  |  |  |  |  |

**Gender**: 1= Male, 2= Female

**Education**: 1= illiterate, 2= Primary Education, 3= Secondary level, 4= SSC, 5= HSC, 6=Bachelor degree. 7= Masters Degree

**Occupation**: 1= Agriculture (crop farming), 2= Poultry/livestock farming, 3= Fisheries, 4= Small business, 5= Govt service, 6= Private Service, 7= Rickshaw/ van pulling, 8= Day labour, 9= Household work, 10= Student, 11= Retired,

- 1. Land Holding

| Land status | Homestead area | Own  cultivated land | Fallow land | Pond Area own | Pond area lease | Mortgage in | Mortgaged out | Total |
| --- | --- | --- | --- | --- | --- | --- | --- | --- |
| Land  in decimal |  |  |  |  |  |  |  |  |

1.3 Asset holding

| Name of assets | Type/No | Value(TK.) |
| --- | --- | --- |
| Television |  |  |
| Radio |  |  |
| Mobile |  |  |
| Refrigerator |  |  |
| Electric fan |  |  |
| Sewing Machine |  |  |
| Motor Cycle |  |  |
| Bi-cycle |  |  |
| Chair table |  |  |
| Agricultural machinery |  |  |
| Total asset value |  |  |

1.4 Livestock Ownership

| Livestock | No | | Value  (TK.) | Sale value of livestock/year  (TK.) | Sale value of livestock product/year  (milk/egg) TK | Amount used for home consumption | |  |
| --- | --- | --- | --- | --- | --- | --- | --- | --- |
|  | Adult | Calf |  |  |  | quantity | value |  |
| Cow |  |  |  |  |  |  |  |  |
| Ox |  |  |  |  |  |  |  |  |
| Goat |  |  |  |  |  |  |  |  |
| Poultry |  |  |  |  |  |  |  |  |
| Total value |  |  |  |  |  |  |  |  |

1.5 Household Debt

Did you take loan? I) Yes II) No

If Yes

| Sources of Loan | code | Amount(TK) | Purpose of taking loan | code | Interest rate/year |
| --- | --- | --- | --- | --- | --- |
| 1 NGO  2 Bank (specify name):  3 Friends  4.Relatives  5.Money lender |  |  | 1.Home expenditure  2. farm inputs buying  3.Marriage  4.Education  5.Health  6.House repair  7.Business  8.Others(specify)  9.No response |  |  |

2 Farm household’s perception on factors influencing migration

| i) Poor living conditions/poverty | Yes/No |
| --- | --- |
| ii) Low wage rate on the rural area compared to urban area |  |
| iii) Lower return from farming |  |
| iv)Unemployment at slack farm period in the rural areas |  |
| v) Inadequate farm land |  |
| vi) Too many family members |  |
| vii) Better job opportunity in urban areas |  |
| viii)Better education facilities in the city |  |
| ix)Attraction to the city/ prospects of enjoying urban facilities |  |
| x) Failure to repay NGO loan |  |
| xi) Migrant relatives influence |  |
| xii) Crop failure due to natural disaster |  |

Place, Date Signature of the Enumerator
